# Leveraging Social Needs Assessments to Eliminate Barriers to Diabetes Self-Management in a Vulnerable Population

**DOI:** 10.3390/ijerph22081213

**Published:** 2025-08-01

**Authors:** Jennifer Odoi, Wei-Chen Lee, Hani Serag, Monica Hernandez, Savannah Parks, Sarah B. Siddiqui, Laura C. Pinheiro, Randall Urban, Hanaa S. Sallam

**Affiliations:** 1 John Sealy School of Medicine (JSSM), University of Texas Medical Branch (UTMB), Galveston, TX 77555, USA; jennifer.o.odoi2@gmail.com (J.O.); rurban@utmb.edu (R.U.); 2 Department of Family Medicine, John Sealy School of Medicine (JSSM), University of Texas Medical Branch (UTMB), Galveston, TX 77555, USA; weilee@utmb.edu; 3 Department of Population Health, Division of Global Partnerships, School of Public and Population Health (SPPH), University of Texas Medical Branch (UTMB), Galveston, TX 77555, USA; hssallam@utmb.edu; 4 MD Anderson Cancer Center, Houston, TX 77555, USA; mhernandez26@mdanderson.org; 5 Department of Patient Services, University of Texas Medical Branch (UTMB), Galveston, TX 77555, USA; sjparks@utmb.edu; 6 Department of Internal Medicine, Division of General Internal Medicine, University of Texas Medical Branch (UTMB), Galveston, TX 77555, USA; sbsiddiq@utmb.edu; 7 Weill Cornell Medical College, New York City, NY 10021, USA; lcp2003@med.cornell.edu; 8 Department of Physiology, Faculty of Medicine, Suez Canal University (SCU), Ismailia 41522, Egypt

**Keywords:** diabetes, intensive diabetes self-management education and support, iDSMES, Social Determinants of Health, social needs assessment, vulnerable population

## Abstract

This article describes the design, methods, and baseline characteristics of the social needs assessment (SNA) of participants enrolled in an ongoing randomized clinical trial implementing a comprehensive approach to improving diabetes self-management and providing an intensive Diabetes Self-Management Education and Support (iDSMES) Program at St. Vincent’s House Clinic, a primary care practice serving resource-challenged diverse populations in Galveston, Texas. Standardized SNA was conducted to collect information on financial needs, psychosocial well-being, and other chronic health conditions. Based on their identified needs, participants were referred to non-medical existing community resources. A series of in-depth interviews were conducted with a subset of participants. A team member independently categorized these SNA narratives and aggregated them into two overarching groups: medical and social needs. Fifty-nine participants (with a mean age of 53 years and equal representation of men and women) completed an SNA. Most (71%) did not have health insurance. Among 12 potential social needs surveyed, the most frequently requested resources were occupational therapy (78%), utility assistance (73%), and food pantry services (71%). SNA provided data with the potential to address barriers that may hinder participation, retention, and outcomes in diabetes self-management. SNA findings may serve as tertiary prevention to mitigate diabetes-related complications and disparities.

## 1. Introduction

The prevalence of diabetes in the United States is slightly above the global prevalence—11.6% (38 M) vs. 11.1% (590 M)—with type 2 diabetes (T2DM) accounting for 90–95% of cases [1,2]. It stands as the eighth leading cause of death in the U.S., which emphasizes its significant morbidity and mortality [3]. Diabetes self-management education and support (DSMES) programs employ an evidence-based approach to provide patients with enhanced self-efficacy, thereby improving health outcomes and enabling them to take control over their chronic condition. These programs have demonstrated efficacy in promoting behavior changes in diet, physical activity, blood glucose monitoring, medication adherence, problem-solving, coping skills, and risk reduction [4]. Clinically, DSMES interventions are associated with significant improvements in hemoglobin A1C and reductions in diabetes-related complications and morbidity. In addition, DSMES has been associated with favorable health outcomes, including weight loss and improved glycemic control [5]. Medicare and most insurers cover diabetes self-management programs, which can be cost-effective in reducing emergency department visits, hospital admissions, and hospital readmissions [6].

The literature demonstrates that the lack of family support, professional guidance, and resources represents barriers to diabetes self-management [7]. Resource scarcity can impede the adoption of a diabetic diet and blood glucose monitoring [8,9]. Case management strategies addressing needs such as housing, finances, and job security have been suggested to improve the likelihood of treatment adherence to self-management goals [5]. Social support from family members, peers, medical professionals, and social programs emerges as a facilitator for diabetes management [10]. Patients who participate are often noted to have increased healthy coping skills, empowerment, and self-efficacy.

In line with the World Health Organization’s holistic definition of health as a “state of complete physical, mental, and social well-being” [11], it is imperative to consider the social, cultural, and economic implications of T2DM, particularly for underserved populations. Factors related to the built environment, such as inadequate access to green spaces for physical activity in urban areas and limited public transportation options for travel to access healthcare and healthy food options, are common challenges that communities face [12]. Silos within healthcare create unnecessary fragmentation between sectors and have even been cited as a driver of poor diabetes self-management [13].

In accordance with the Agency for Healthcare Research and Quality’s (AHRQ) Plan-Do-Study-Act (PDSA) method [14], the social needs assessment (SNA) is a tool for identifying the Social Determinants of Health (SDH) that can hinder diabetes self-management efforts. A conceptual hierarchy of social needs for people with diabetes has been detailed elsewhere [15]. Studies assessing the social needs of people with diabetes in metropolitan areas have identified at least one social need; however, for the underserved population, we anticipate a greater need.

This study implemented the intensive Diabetes Self-Management Education and Support (iDSMES) Program as part of a randomized clinical trial to investigate the effects of a comprehensive approach to diabetes self-management in a vulnerable population in Galveston. The comprehensive approach was believed to improve clinical health outcomes among people with type 2 diabetes. The study protocol was approved by The University of Texas Medical Branch (UTMB)’s Institutional Review Board, and written consent was obtained from all subjects prior to enrollment in this study. It is deemed a minimal risk study. The study is registered in clinicaltrials.gov (Identifier: NCT05097534—22 September 2021). The trial is still ongoing. No patients or public were involved in the design, conduct, and reporting of this trial. No protocol change was made after its commencement. This section of the clinical trial aimed to analyze the SNA of study participants and identify barriers to diabetes self-management.

## 2. Materials and Methods

**Design, Participants, and Sample Size:** The clinical trial was conducted at St. Vincent’s House Clinic, a faith-based primary care practice serving vulnerable and economically and resource-challenged populations in Galveston. In addition to healthcare services, the clinic offers a range of social services, including a food pantry, homebound grocery delivery, homelessness prevention and emergency assistance, transportation, and medical care. The clinic also partners with various community-based organizations to offer vision care, dental care, speech therapy, occupational therapy, physical therapy, and mental health counseling. Most services are provided free of charge; only a few services charge patients a small co-payment or require advance appointment scheduling. Patients who visit the clinic are predominantly low-income and under- or uninsured. The clinic sees an average of 2000 patients annually, with approximately 28% having diabetes.

Utilizing electronic medical records, 150 eligible individuals with T2DM were identified and recruited to the clinical trial through phone calls, emails, and provider referrals, following approval from the clinic director, thereby avoiding cold calls. Eligibility criteria included being 18–84 years of age and having type 2 diabetes with an A1C > 7% (Table 1). Medical and public health students were trained to contact and recruit eligible participants, obtain their consent, and collect data for the clinical trial. Identified people with T2DM visited the clinic, where medical students noted their consent, conducted biometrics measurements such as weight, height, BMI calculation, blood pressure, Point-of-Care hemoglobin A1C (A1C), and lipid profile, and collected baseline survey data on demographics, socioeconomic status, dietary and activity habits, quality of life, and medication adherence under faculty supervision. Participants were randomized to receive either the standard of care or the intervention (i.e., a comprehensive approach), which included the iDSMES program and provision of social and other health services, including physical therapy, access to the food pantry, and nutritional consultation. A parallel group design was chosen to explore the superiority of the comprehensive approach vs. the standard of care. The outcomes of the clinical trial are presented elsewhere. This article focuses on the process of SNA.

To offer individualized social services, all participants in the intervention arm were referred to the social worker for SNA. We used the standardized CMS tool, administered by an English-speaking, licensed social worker, on the day of the consent visit. A Spanish-speaking student volunteer translated questions and responses for Spanish-speaker participants. The social worker collected information on each participant’s financial, social, physical, and mental health conditions. Participants were asked about (1) their income sources and whether they need assistance reducing their financial burdens, (2) mobility, balance, and muscle strength to identify potential needs for physical and occupational therapies, and (3) mental health history, psychotropic medications, and psychiatric care. Given the individualized nature of participant needs, the social worker assessed each case and facilitated referrals to necessary agencies and programs. All participants were referred to occupational therapy for a baseline assessment of functioning and participation in activities of daily living (ADLs). Most educational materials and resource guides were translated into Spanish to maximize accessibility.

**Figure 1:**
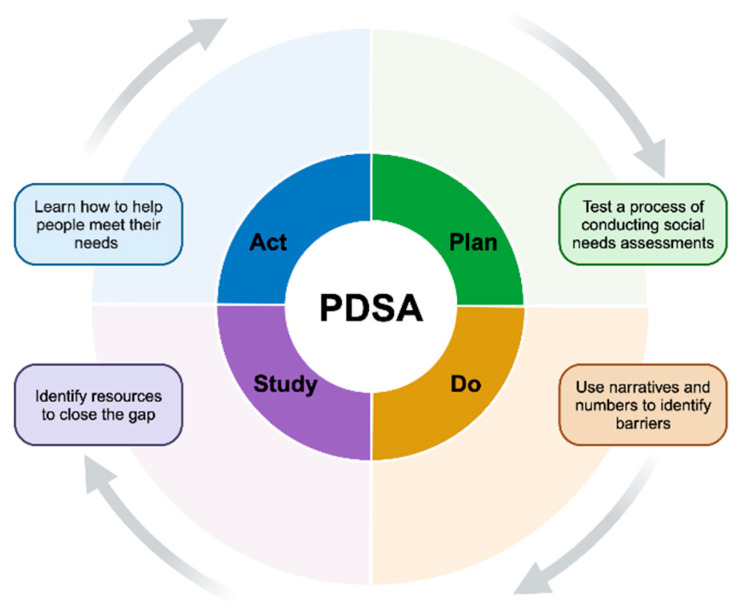
Social Needs Assessment Study Framework According to the Plan-Do-Study-Act (PDSA) Method.

**Ethical considerations:** The SNA was conducted in a private setting through narrative interviews lasting two hours, allowing the investigators to provide valuable resources to meet the social needs of participants, as per the PDSA method (Figure 1).

**Variables:** Participants’ responses to the SNA were transcribed. The demographics section consisted of the participants’ age, sex, race, marital status, household size (including the participants themselves), and whether they were caregivers for others. Responses were categorized as follows: (a) socioeconomic status section focused on participant income, insurance coverage, housing situation, transportation options, utility access, and participation in social welfare programs such as Temporary Assistance for Needy Families (TANF) and Supplemental Nutrition Assistance Program (SNAP), and (b) health section captured data on physical, mental, and behavioral concerns. No missing data or refusals were observed by this group of completers. Based on these responses, the final section reports the resources recommended by the social worker.

Social needs were coded as follows: each type of need was assigned a value of 1, indicating a need for assistance, or 0, indicating no assistance was required. Participant needs were aggregated to obtain an overall score from 0 to 12, with a higher score indicating a greater number of needs. Coding the social needs as binary allowed us to identify the barriers to diabetes self-management, enabling resources to be tailored to address these barriers. The number of participants who needed assistance was also calculated to obtain an overall headcount from 0 to 59, with a higher count indicating a greater number of participants requiring that specific type of assistance.

**Data Analysis:** A descriptive analysis of the SNA data was conducted to present participant characteristics and their SNA scores. A team member independently categorized these SNA narratives and aggregated them into two overarching groups: medical and social needs. Frequency and percentage calculations were used to compare and identify primary social needs. Excerpts from social workers’ notes were also reported qualitatively for each type of assistance.

## 3. Results

A total of 59 participants completed the SNA with the social worker between January 2022 and March 2023. Table 2 demonstrates the characteristics of completers. The average age of completers was 53 years (±1.4). Most completers identified themselves as Hispanic/Latino (63%). Over half were female (53%). Approximately two in five participants (41%) were married or had partners, and half (51%) reported being caregivers for their family members. More than 70% of participants reported lacking health insurance, and 1 in 4 participants expressed concerns related to behavioral and mental health (25%). Participants reported that the mean monthly salary was $1449 (±1314), with 17 participants (29%) reporting a monthly income of $0.

Table 3 shows the quantity and variety of needs reported by participants. Only four participants did not report any social or healthcare-related needs at the time of assessment. More than 93% of participants had at least one need. The average number of needs per participant was 4.3. There were 19 additional types of needs, each reported by fewer than 10 participants. Of the 17 participants who had insurance, 15 (88%) reported needs during their SNA. When stratifying participants by age (<65 and ≥65 years), we observed that the younger group (*n* = 51), on average, had 4.51 needs, whereas the older group (*n* = 8) had only 2.75 needs (*p* = 0.048). We repeated the same analysis for other characteristics, such as gender, race, marital status, and income. However, none of these are significantly different in the number of social needs (*p* > 0.05).

**Figure 2:**
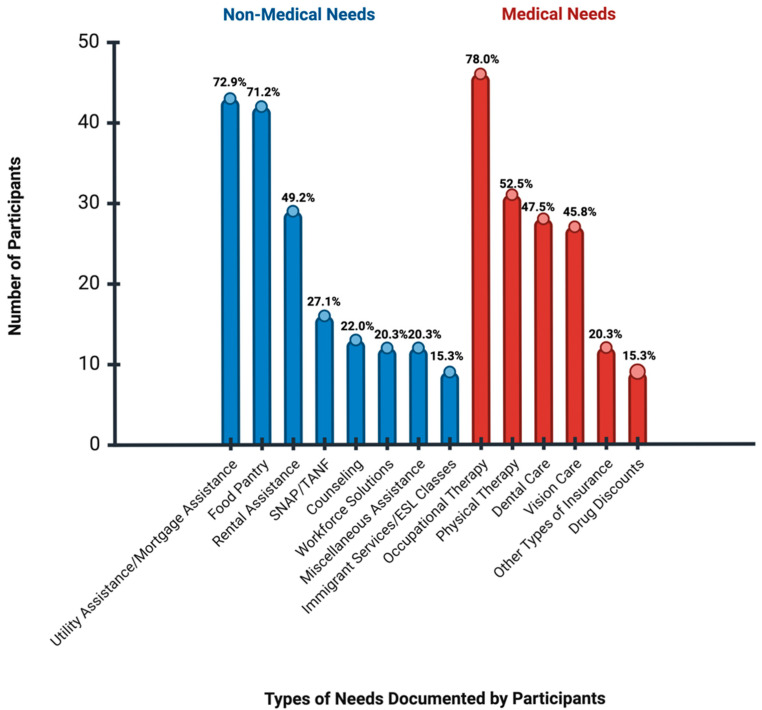
Ranking of Needs Expressed by Participants. More than 45 participants reported healthcare-related needs. Over 40 participants mentioned their needs for utility assistance and food pantry, which is four times more than the number of participants demanding workforce solutions.

Figure 2 demonstrates the ranking of needs expressed by participants, with the highest percentage of individuals needing occupational therapy (78%), followed by utility assistance (73%). The three most frequently reported needs were as follows.

### 3.1. Occupational Therapy

All participants were asked about whether (a) they have any difficulty with using their hands, (b) if they have fallen in the past two months, (c) if they can reach their feet or (d) if they find themselves feeling tired frequently. Based on their responses, 46 were referred to occupational therapy offered through the clinic.

“He reports chronic pain in his left leg and lower back stating that it’s more intense when he is sitting or lying down. He occasionally experiences numbness and tingling in his hands, and it has also started in his feet. He also reports having the same tingling sensation in his face from time to time which he has addressed with his PCP.” (ID#36, male, 42 years old, extracted from social worker SNA summary).

### 3.2. Utility Assistance:

While some participants required rent assistance (48%), 73% expressed interest in applying for the utility assistance program.

“She owns her mobile home and pays $400 a month, which includes water, to rent the lot. She pays for electricity and gas and is open to applying for the Baker Ripley Utility Assistance Program.” (ID#29, female, 50 years old, extracted from social worker SNA summary)

### 3.3. Food Insecurity

Many participants expressed difficulty completing SNAP applications. As a result, the social worker assisted them with filling out applications and referred them to food pantry services available at St. Vincent’s Hope Clinic. Participants were also referred to navigators who assisted them with applying for SNAP.

“She reported that they applied for SNAP in March but were denied. She expressed some confusion with HHSC correspondence, but believes they were denied because of her income. She would like assistance with reapplying as she believes they should be eligible with her husband who is not working. She reported that they do not utilize food pantries at this time and are experiencing food insecurity.” (ID#81, female, 42 years old).

Following SNA, resources are identified to help participants overcome barriers to diabetes self-management. The social worker linked the participants to appropriate resources based on their needs (e.g., occupational therapy assessments and plans, utility assistance resources, access to a diabetes-friendly food pantry, and SNAP application assistance).

## 4. Discussion

This project identified selected SDH barriers in participants enrolled in a clinical trial to investigate the effects of a comprehensive approach to diabetes self-management in a socioeconomically disenfranchised population in Galveston. In a group of 59 participants comprising middle-aged, primarily Hispanic uninsured females, a standardized SNA revealed the following: (1) More than 93% of participants had at least one need, with an average of 4.3 needs per participant. (2) Even participants who had credible coverage such as Medicare or Medicaid had needs. (3) Occupational therapy ranked first as a need, followed by utility assistance, then food insecurity.

### 4.1. Medical Needs

Approximately 93% of participants demonstrated at least one healthcare-related need. The most demanding need was for occupational therapy, expressed by 78% of participants. These needs may stem from challenges associated with suboptimal disease management and the progression of diabetes, its complications, and comorbidities. Since 71% of participants were uninsured, they likely lacked consistent access to medical services. The need for physical therapy, as reported by 53% of participants, is crucial for promoting physical activity, a key component of diabetes self-management.

As expected, dental and vision care remain highly sought after in this study population, given the diabetes-related ophthalmological and dental complications [16,17]. However, adult preventive dental care is not covered by Medicaid or traditional Medicare. With people with diabetes experiencing multiple comorbidities, prioritization and management of medical conditions, including diabetes, becomes challenging [18]. Therefore, dental and vision care are essential for maintaining health, preventing complications, and treating diseases.

Approximately 25% of participants expressed mental health or behavioral concerns. This is conceivable as people with diabetes are more likely to have depression and anxiety than those who do not have chronic diseases [19].

These medical conditions may directly impact participants’ self-efficacy and empowerment in their diabetes self-management. For example, after realizing that SNA may need to be expanded to evaluate and refer participants to mental health services, such increased investment into specialty services will allow recognition of the full spectrum of health. Additionally, SNA illuminated participants’ interest in medication discounts and vouchers to mitigate the high costs charged by external pharmacies. Insured participants may only have access to screening tests annually, and the frequency may decrease for uninsured participants. Other considerations include increasing access to monitoring tests, such as hemoglobin A1C and lipid panels, to ensure that participants are aware of the effectiveness of their diabetes management and glycemic control.

### 4.2. Social Needs

The substantial need for rental, mortgage, and utility assistance (73%) demonstrates the influence of non-medical factors on diabetes self-management. It is known that a sense of security and control in one’s home environment can promote diabetes self-management [8]. Unstable housing has been linked to increased utilization of emergency department care and hospitalization for diabetes-related complications [20]. Housing and utility costs are often highly prioritized expenses [21]. Therefore, diverting financial resources towards these necessities may increase the strain to afford facilitators of diabetes self-management, such as purchasing fresh produce.

Food insecurity was prevalent among this study population, with most participants (71%) citing a need for food pantry services. Food insecurity can limit opportunities to exercise self-efficacy in food choice, often resulting in diets that are more carbohydrate-dense and have higher glycemic loads, which can adversely affect diabetes self-management and control. Despite the significant need for food pantry services, only 25% of participants currently participate in SNAP. Many of the participants in the study who did not seek SNAP were of non-residency status, which may be a potential barrier to qualification. National, state, and local policies influence food insecurity. SNA may be used as a bridge to explore unique strategies for meeting participant needs, such as food box deliveries [22].

### 4.3. Lessons Learned

**Schedule Limitations:** SNA necessitates a thorough evaluation of the SDH and available resources, making it a time-intensive process. Since the availability of our social worker and student volunteers may be limited at times, scaling up study practices could help meet the needs of the participant population and increase sustainability. Recruitment of student volunteers to be trained to conduct SNA may allow for more varied schedule options, including after-hours sessions to accommodate participant work schedules. However, student volunteers may also have limited time due to academic requirements. Integrating value-added experiential learning into the healthcare professional curriculum can help streamline the process with the SNA evaluation, while also training future healthcare professionals in these crucial skills. Additionally, increasing personnel can enable multiple SNAs to be conducted simultaneously, thereby enhancing efficiency.

**Language Barriers:** Approximately 63% of the participants identified as Hispanic/Latino, with many requiring SNA to be conducted in Spanish. Offering translation services or Spanish-speaking volunteers can facilitate faster and more effective communication. Additional obstacles may be encountered if application resources are not offered in other languages.

**Transportation Barriers:** SNA is not traditionally covered by insurance, making grant funding essential for long-term sustainability. Competing priorities related to health and finances may hinder access to and utilization of social services [21]. While only 11% of participants initially expressed being without transportation, transportation proved to be a constraint that prevented participants from accessing the study. Many participants also lost transportation as they progressed through the study, and they often required rescheduling their appointments due to unreliable transportation. Offering bus tokens and cab vouchers was crucial in addressing this issue. However, transportation limitations, including limited bus routes and availability, may persist, which can affect participants’ ability to access resources and attend appointments. Bus routes may also present challenges for participants with low literacy or who do not speak English. Some participants lacked childcare and desired to bring their children to appointments; however, not all cab services offered appropriate car seats for children or were accessible to participants with disabilities. Transportation barriers may also affect their ability to access resources, such as free medications provided by the St. Vincent’s Clinic pharmacy.

### 4.4. Strengths and Limitations

The strengths of this study lie in its emphasis on the importance of a motivating health system that provides a more comprehensive SNA, ensuring participants are linked to necessary resources. SNA is the first step in the PDSA Method (Figure 1), by which we identified barriers to diabetes self-management for participants. In this ongoing clinical trial, resources are identified to assist participants in closing this gap and reducing or removing the barriers, thus helping them to meet their needs.

Additionally, this study aims to empower participants with self-efficacy to make informed, healthy decisions. With the average number of needs within this study population exceeding 4, coordinating applications for various services may become burdensome. The extensive application processes that many social services require can serve as additional barriers to accessing these services. Government assistance programs are budgeted and administered by multiple agencies, posing navigation challenges for participants with low literacy. Many organizations share a mission to improve chronic disease management by addressing social needs by integrating an SDH screening tool into their electronic medical records. Potential solutions include addressing transportation needs through participant navigation programs, building relationships with community organizations, and increasing advocacy at both federal and state levels [23].

This study was conducted at a free student-run clinic in Galveston, Texas, serving primarily uninsured and underinsured participants. As such, generalizability is limited. The relatively small sample size also precluded the investigators from estimating multivariable models [24].

## 5. Conclusions

Participants enrolled in this clinical trial indicated a high level of social needs. SNA provided data with the potential to address barriers that may hinder participation, retention, and outcomes in diabetes self-management. SNA findings may serve as a tertiary prevention to mitigate diabetes-related complications and disparities.

## Figures and Tables

**Table 1:** Participation Criteria for a Clinical Trial Investigating the Effects of a Comprehensive Approach to Diabetes Self-Management in a Vulnerable Population in Galveston.

Inclusion Criteria	Exclusion Criteria
18–84 years of age; Have type 2 diabetes; Hemoglobin A1C > 7%.	Cognitive impairments prohibiting informed consent participation, as indicated by the referring physician Current drug abuse Undergoing active treatment for cancer Inability to participate in the 12-month duration intervention (e.g., disorders that might compromise survival, planning to relocate outside Galveston Island)

**Table 2:** Characteristics of Participants in a Clinical Trial Investigating the Effects of a Comprehensive Approach to Diabetes Self-Management in a Vulnerable Population in Galveston.

Item	Participants (*N* = 59)
**I. Demographics**	
Age (mean, std.)	53.47 (1.40)
Male (*n*)	28
Female (*n*)	31
*Race & Ethnicity* (*n*)	
Non-Hispanic Black	11
Non-Hispanic White	11
Hispanic/Latino	37
*Marital Status* (*n*)	
Single, Widowed, Divorced, Separated	35
Married or With Partner	24
Number of Household Members (mean, std.)	2.93 (0.19)
*Being Caregiver* (*n*)	
None	29
Care for children or grandchildren	25
Care for spouse or parents	5
**II. Socioeconomic Status**	
Monthly Salary (mean, std.)	$1449 ($1314)
Without Insurance (*n*)	42
Without SNAP * (*n*)	41
Housing by Renting (*n*)	38
Without Transportation (*n*)	11
Without Utilities (*n*)	7
Have Activities of Daily Living (ADL) Limitations (*n*)	8
Have History of Substance Abuse (*n*)	6
Have Behavioral/Mental Health Concerns (*n*)	15

* SNAP: Supplement Nutrition Assistance Program.

**Table 3:** Social Needs Assessment Scoring Among Participants in a Clinical Trial Investigating the Effects of a Comprehensive Approach to Diabetes Self-Management in a Vulnerable Population in Galveston.

Service Needs	Participants (*N* = 59)
No. of Needs (max, min)	0–9
No. of Needs (mean, std.)	4.27 (0.31)
**No. of Needs (*n*, %)**	
0	4 (6.8%)
1–2	11 (18.6%)
3–4	15 (25.4%)
5–6	21 (35.6%)
7+	8 (13.6%)

## Data Availability

The original contributions presented in this study are included in the article. Further inquiries can be directed to the corresponding author.

## Abbreviations

The following abbreviations are used in this manuscript:

ADL	Activities of Daily Living
AHRQ	Agency for Healthcare Research and Quality
DSMES	Diabetes Self-Management Education and Support
iDSMES	Intensive Diabetes Self-Management Education Program
PDSA	Plan-Do-Study-Act
SDH	Social Determinants of Health
SNA	Social Needs Assessments
SNAP	Supplemental Nutrition Assistance Program
T2DM	Type 2 Diabetes Meletus
TANF	Temporary Assistance for Needy Families
UTMB	University of Texas Medical Branch
